# Paediatric monkeypox patient with unknown source of infection, the Netherlands, June 2022

**DOI:** 10.2807/1560-7917.ES.2022.27.29.2200552

**Published:** 2022-07-21

**Authors:** A Marceline Tutu van Furth, Martijn van der Kuip, Anne L van Els, Lydia CR Fievez, Gini GC van Rijckevorsel, Anton van den Ouden, Marcel Jonges, Matthijs RA Welkers

**Affiliations:** 1 Amsterdam UMC location Vrije Universiteit Amsterdam, Emma Children’s Hospital, Department of Paediatric Infectious Diseases and Immunology, Amsterdam, The Netherlands; 2 Amsterdam Institute for Infection and Immunity, Infectious diseases, Amsterdam, The Netherlands; 3 Public Health Service Amsterdam, Dept. of Infectious Diseases, Amsterdam, The Netherlands; 4 Centre for Infectious Disease Control, National Institute for Public Health and the Environment (RIVM), Bilthoven, The Netherlands; 5 Molecular Biology Systems BV, Goes, The Netherlands; 6 Amsterdam UMC location AMC, University of Amsterdam, Department of Medical Microbiology and Infection Prevention, Amsterdam, The Netherlands; 7 Public Health Service Amsterdam, Public Health Laboratory, Amsterdam, The Netherlands

**Keywords:** Monkeypox, child, Netherlands, whole genome sequencing

## Abstract

Since May 2022, an international monkeypox (MPX) outbreak has been ongoing in more than 50 countries. While most cases are men who have sex with men, transmission is not restricted to this population. In this report, we describe the case of a male child younger than 10 years with MPX in the Netherlands. Despite thorough source tracing, a likely source of infection has not been identified. No secondary cases were identified in close contacts.

While repeated community outbreaks of monkeypox (MPX) have been reported in African countries and the United States, these were mainly caused by spillover events from animals to humans [1,2]. Many of these cases also involved children, with a case fatality rate (CFR) between 3.6% and 10.6% depending upon the MPX clade [3,4]. The current global outbreak of MPX does not appear to have a clear link to Africa and distinguishes itself from previous reported outbreaks in that there is more sustained transmission within the community of men who have sex with men (MSM) [5]. The infection of children is very rare and warrants further investigation. 

## Clinical case description

**Figure 1:**
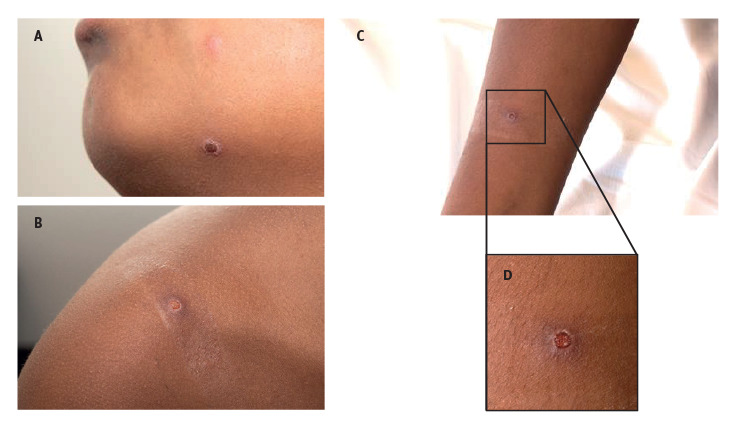
Monkeypox lesions, paediatric patient, the Netherlands, June 2022

At the end of June 2022, a male child younger than 10 years without relevant medical history presented at a paediatric emergency room (ER) in Amsterdam, the Netherlands. He was vaccinated according to the Dutch national vaccination programme and had chicken pox when he was 5 years-old. Three weeks before his visit, he experienced a sore throat without fever that spontaneously resolved on the next day. A day later, he travelled to Turkey for a 1-week holiday. After his return, he noticed two small round skin lesions on his left lower jaw and cheek (Figure 1A). The general practitioner (GP) started with antifungal cream under the suspicion of a mild dermatomycosis. In the following days, more lesions appeared in the child’s face. The GP was again consulted and antibiotic cream was started for suspected impetigo vulgaris. When ca 20 solitary lesions appeared on other body parts (Figure 1B-D) the child was referred to our hospital under the clinical suspicion of MPX. On physical examination, we observed an alert child in overall good health with stable vital parameters and without fever. There were no enlarged lymph nodes in the neck, armpits or groin region. The liver or spleen did not seem enlarged upon abdominal palpation. On the skin, we observed a centrifugal distribution of 20 solitary, sharply demarcated, red-brown vesicles (left ear, left lower jaw, both forearms, both thighs and on the back). No lesions in the oral cavity or genital region were seen.

Panel A: two solitary lesions on the left lower jaw and cheek; Panel B: right shoulder; Panel C: right forearm; Panel D: forearm zoomed in. Pictures were taken 10 days after appearance of the first lesion. Published with parental consent.

Laboratory analysis showed a normal erythrocyte sedimentation rate (12 mm/h; reference value: 0–13 mm/h) and low C-reactive protein (1,9 mg/L; reference value: 0–5 mg/L). Haemoglobin, thrombocytes and leucocyte counts were also in the normal range. Unexpectedly, the child had an immunoglobulin A (IgA) deficiency (0.45 g/L; reference value: 0.53–2.04 g/L). 

As the major transmission route in the current MPX outbreak is related to sexual activity [5], we ruled out the possibility of sexual abuse by taking careful history and tested for syphilis (negative serology), gonorrhoea (negative peri-anal swab PCR), chlamydia (negative peri-anal swab PCR), HIV infection (negative P24 antigen/antibody test on serum) and hepatitis B and C (negative HBsAg, negative anti-HB core, positive anti-HbS, negative anti-HCV). A PCR for varicella zoster virus on the vesicle fluid was also negative.

**Figure 2:**
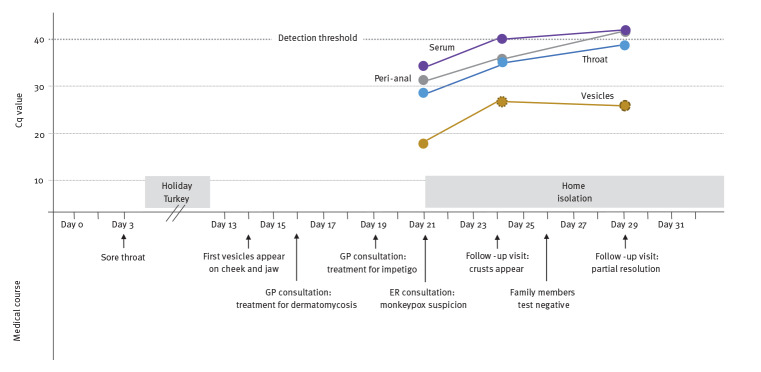
Timeline of key medical observations related to PCR quantification cycle values, paediatric monkeypox patient, the Netherlands, June 2022

**Figure 3:**
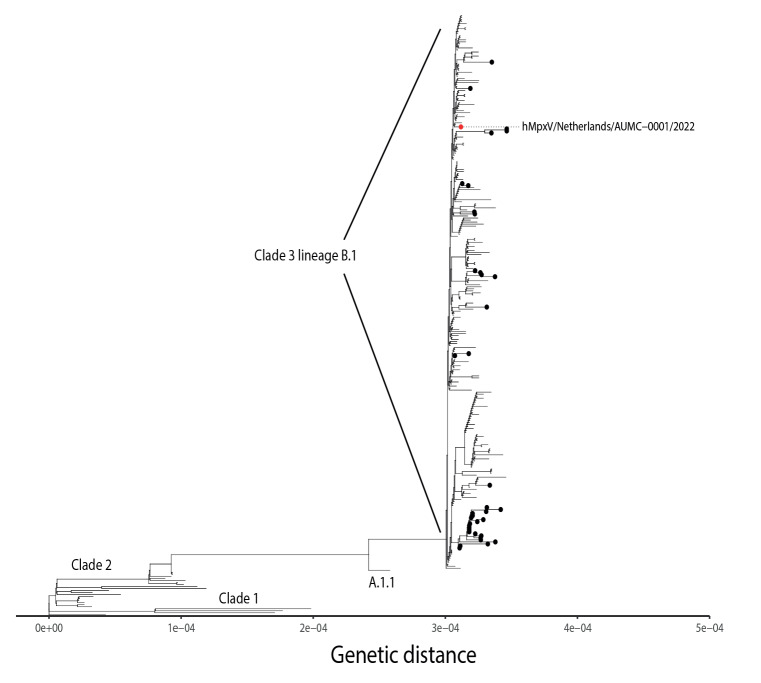
Whole genome sequencing of monkeypox virus from vesicle fluid, paediatric patient, the Netherlands, June 2022

Samples for MPX virus (MPXV) PCR testing were taken from blood, throat, anal region, skin vesicles and urine [6,7]. See the Supplement for details concerning diagnostic tests. All obtained samples except for the urine tested positive for MPXV. The PCR quantification cycle (Cq) values and key medical observations are shown in Figure 2. Whole genome sequencing of the vesicle fluid showed that the patient’s sequence clustered within the clade 3 lineage B.1 (Figure 3). The sequence has been deposited in GISAID (EPI_ISL_13728303) with strain name hMpxV/Netherlands/NH-AUMC-0001/2022; details on sequencing are provided in the Supplement. This B.1 lineage is responsible for the current MPX outbreak in Europe [8]. The absence of any shared genomic transmission markers hampered the reconstruction of a specific transmission chain. Family members (the parents and two siblings) tested PCR-negative for MPXV in serum, urine, throat and peri-anal region and in all skin swabs obtained from potential lesions.

Cq: quantification cycle; ER: emergency room; GP: general practitioner.

The Cq value of the throat swab increased over time, indicating a potentially reduced risk of respiratory transmission. The clinical course of our patient showed fast resolution of the pox into crusts, no systemic signs of infection and no new lesions. Nevertheless, the Cq value of the vesicles remained relatively low (Cq = 27 on Day 29). It is unclear whether this reflects active replication of infectious virus.

MPX: monkeypox.

Phylogenetic tree visualising available MPX virus sequences from the clades 1, 2 and 3. Highlighted in red: the described case which clusters within the clade 3 B.1 lineage, responsible for the current outbreak. Black dots: sequences obtained from epidemiologically unrelated MPX cases identified in the Amsterdam region in 2022.

## Public health response

In the Netherlands, MPX has been a notifiable disease according to Dutch law since 21 May 2022. Source and contact tracing was initiated immediately upon confirmation of the diagnosis. An outbreak team convened at the Public Health Service (PHS) Amsterdam, consisting of representatives of the PHS, clinicians and a medical microbiologist. The team executed the risk assessment, risk classification and control measures (including isolation) regarding the patient and his contacts and coordinated the risk communication together with experts of the Dutch Centre for Communicable Diseases. Extensive source and contact tracing did not identify a potential source. The child had not been in contact with persons with a proven or possible MPXV infection. During their holiday in Turkey, the family had consistently covered the bed chairs at the pool with their own towels and there was no close contact with other guests.

Contacts that were identified as high-risk contacts (parents, a friend and one sibling) promptly received the smallpox vaccine Imvanex. Letters were sent by the PHS to the school and a sports club that the patient attended during the infectious period. Contacts at the school and the sports club were classified as no-risk or low-risk contacts and were requested to report to the local authorities for testing in case they experienced symptoms matching a possible MPXV infection. No secondary cases have been identified.

## Discussion

We describe a paediatric case of MPX in the Netherlands. We were not able to identify any possible source of the infection. Whole genome sequencing positioned the patient’s sequence within the clade 3 lineage B.1, but did not directly link to any other strains from the Amsterdam region.

As no plausible source could be identified, this leaves us with an open question regarding transmission. In the current outbreak, the predominant route of transmission is related to sexual activity in the community of men who have sex with men [5]. However, other indirect transmission routes have been described, such as respiratory transmission through droplets or contaminated materials such as bedding and towels [3,4,9,10]. Therefore, it is possible that the child was in close contact with an infectious person or contaminated object that was not recognised as such. While the described incubation can vary between 5 and 21 days, the estimated mean incubation period within confirmed patients in the Netherlands has been estimated at 8.5 days [11]. This would indicate infection in the beginning of June 2022. However, this may be inaccurate as the route of transmission in this case was different, which in turn may have increased the incubation period. Coincidentally, the patient was also diagnosed with an IgA deficiency. These patients are prone to sinopulmonary infections because of impaired mucosal immunity. Considering that IgA neutralises the virus at the mucosal level, this suggests that respiratory transmission may have played a role. Many facts of the current MPX outbreak are unknown, such as the contagious period. The virus was well detectable in the upper airway and intact peri-anal skin, without any visible pox lesions at these regions, but decreased to undetectable levels within a week. The fluid in the resolving vesicles remained PCR-positive with a low Cq value 3 weeks after the start of symptoms, indicating the possible presence of replication-competent virus. Fortunately, we have not detected any secondary cases related to the patient. 

## Conclusion

With this case description we wish to raise awareness among clinicians that MPX can develop in children and be present in the general population. We advise prompt diagnostic testing in case of clinical symptoms potentially related to MPX to prevent potential undetected transmission in the community. We advocate vaccination for high-risk contacts to prevent potential disease and successive transmission.

## Supplementary Data

129000 Supplement
