# Supplementary material for: Paediatric monkeypox patient with unknown source of infection, the Netherlands, June 2022
Source: Euro Surveill. 2022 Jul 21;27(29):2200552. doi: 10.2807/1560-7917.ES.2022.27.29.2200552 (PMC9306258; doi:10.2807/1560-7917.ES.2022.27.29.2200552)
Supplement: Supplement [file 22-00552_WELKERS_Supplement.pdf]

This supplementary material is hosted by *Eurosurveillance* as supporting information alongside the article "Paediatric monkeypox patient with unknown source of infection, the Netherlands, June 2022" on behalf of the authors who remain responsible for the accuracy and appropriateness of the content. The same standards for ethics, copyright, attributions and permissions as for the article apply. Supplements are not edited by *Eurosurveillance* and the journal is not responsible for the maintenance of any links or email addresses provided therein.

## Molecular diagnostics

Molecular MPXV diagnostics was performed using two (F3L and G2R) MPXV specific targets in a duplex real-time PCR assay with internal amplification controls. Both F3L and G2R primer and probe sequences were obtained from literature, but the assays were in-house modified and validated to run on FLOW lab automation (Roche Diagnostics International, Rotkreuz, Switzerland)[1,2].

## Whole genome sequencing and analysis

Whole genome amplification was performed using primer sequences developed and kindly provided by Martin Schou Pedersen (Department of Clinical Microbiology, Rigshospitalet, Copenhagen University Hospital, Copenhagen, Denmark). In short, all gene segments of the monkeypox virus genome were amplified using a PCR tiling approach. Two separate PCR reactions (pool A/B) were performed for each monkeypox positive sample. Each reaction contains 44 primers generating 2500 bp amplicons. PCR tiling was performed using a 42-minute PCR program on a NextGenPCR thermal cycler (MBS, The Netherlands). After the multiplex PCR, purification was performed using the AMPure XP beads (Beckman Coulter, CA, USA). Library quantification was performed by using Qubit dsDNA HS assays kits (Thermo Fisher, catalog nr. Q32854) After normalization each individual sample was barcoded with the Oxford Nanopore SQK-RBK110.96 rapid barcoding kit. The samples were then pooled together in a single tube and again purified using AMPure XP beads (Beckman Coulter, CA, USA), samples were then processed based on the manufacturer's protocol. Oxford Nanopore sequencing was performed by on a Nanopore Minion R9 flowcell using high accuracy basecalling.
A detailed step-by-step protocol is available on protocols.io (https://www.protocols.io/private/974ACB78F83911EC998C0A58A9FEAC02).

Raw fastq sequencing data was initially mapped to the human genome hg19 to remove contaminant human reads using minimap2 version 2.18-r1015 with default settings[3]. Unmapped reads were remapped against reference sequence MPXV_USA_2022_MA001 (Genbank accession number ON563414.2). The resulting sam-file was coverted to bam-file format, sorted and indexed using samtools v.1.10[4]. A consensus sequence was generated using the TrueConsense package (https://github.com/RIVM-bioinformatics/Trueconsense) using settings (-cov 30 -noambig). Resulting consensus sequence of the described case was deposited in GISAID with EPI_ISL_13728303 with strainname hMpxV/Netherlands/NH-AUMC-0001/2022. Additional hMPX sequences were obtained via mpox-spectrum.org (https://mpox.genspectrum.org/) by downloading the available pre-aligned sequences and combined with the obtained sequence of this case. Phylogenetic analysis was then performed using the nextstrain[5] monkeypox build (https://github.com/nextstrain/monkeypox).

Phylogenetic tree was rooted to the sequence of Genbank accesion number MK783032 and visualized using the ggtree2 package in R version 4.0.3 [6,7].

## References


1. Maksyutov RA, Gavrilova E V., Shchelkunov SN. Species-specific differentiation of variola, monkeypox, and varicella-zoster viruses by multiplex real-time PCR assay. J Virol Methods [Internet] 2016;236:215–20. Available from: http://dx.doi.org/10.1016/j.jviromet.2016.07.024
2. Li Y, Zhao H, Wilkins K, Hughes C, Damon IK. Real-time PCR assays for the specific detection of monkeypox virus West African and Congo Basin strain DNA. J Virol Methods 2010;169(1):223–7.
3. Li H. Minimap2: Pairwise alignment for nucleotide sequences. Bioinformatics 2018;34(18):3094–100.
4. Li H, Handsaker B, Wysoker A, et al. The Sequence Alignment/Map format and SAMtools. Bioinformatics [Internet] 2009 [cited 2011 Jul 5];25(16):2078–9. Available from: http://www.pubmedcentral.nih.gov/articlerender.fcgi?artid=2723002&tool=pmcentrez&rendertype=abstract
5. Hadfield J, Megill C, Bell SM, et al. NextStrain: Real-time tracking of pathogen evolution. Bioinformatics 2018;34(23):4121–3.
6. R Development Core Team. R: A language and environment for statistical computing [Internet]. 2008;Available from: http://www.r-project.org
7. Yu G, Smith DK, Zhu H, Guan Y, Lam TTY. Ggtree: an R Package for Visualization and Annotation of Phylogenetic Trees With Their Covariates and Other Associated Data. Methods Ecol Evol 2017;8(1):28–36.
